# Who Controls the Prosthetic Leg? Voluntary Control Beyond the Microprocessor Knee

**DOI:** 10.7759/cureus.113215

**Published:** 2026-07-23

**Authors:** Mitsunori Toda

**Affiliations:** 1 Orthopedic Surgery, Rehabilitation Medicine, Hyogo Prefectural Rehabilitation Center Central Hospital, Kobe, JPN

**Keywords:** body schema, embodiment, gait, human-device coordination, lower limb prosthesis, microprocessor-controlled knee, motor learning, prosthetic rehabilitation, transfemoral amputation, voluntary motor control

## Abstract

Microprocessor-controlled knees (MPKs) have meaningfully advanced the safety and confidence of many transfemoral prosthesis users, yet a persistent clinical paradox remains: improved device intelligence does not automatically translate into greater everyday activity or full functional recovery. This editorial argues that the value of an advanced prosthetic leg is realized not when the device assumes control on the user's behalf, but when it is coupled with the user's own voluntary control and human-device coordination. Drawing on evidence that objective activity does not consistently increase after MPK provision, that hip strength alone poorly predicts walking and balance, and that attention to the prosthesis increases with use, I propose that rehabilitation should invest in the human control loop as deliberately as in device technology. The microprocessor knee serves here as the representative case for a broader principle that may plausibly extend to microprocessor feet, hip disarticulation components, and powered limbs, though this extension is offered as a hypothesis for future investigation rather than an evidence-based conclusion. My organizing proposition is that, in the age of intelligent prostheses, the human remains the origin of control.

## Editorial

The benefits of the microprocessor knee are real

There is little serious dispute that microprocessor-controlled knees (MPKs) benefit their users. A systematic review by Kannenberg and colleagues concluded that even limited community ambulators derive meaningful gains from MPKs, including fewer falls and stumbles, greater confidence, and improved performance on functional tasks compared with non-microprocessor knees [1]. The MPK serves here as the representative example of the wider class of microprocessor and powered prostheses, precisely because its evidence base is the most mature. It is important, however, to be clear about what these benefits are and are not. The device processes sensor input and modulates stance and swing resistance to reduce the consequences of imperfect control. That is not the same as the device taking over control from the user. In what follows, I refer to the device's sensing and resistance modulation as the device-side control loop, and to the user's voluntary muscular control and postural coordination through the socket as the human-side control loop. This distinction -- easy to blur when a prosthesis performs impressively -- sets up the paradox that follows.

A paradox: better devices, unchanged activity

If a more capable knee straightforwardly produced a more active person, we would expect objective activity to rise after provision. In at least one recent study, it did not. Caggiari and colleagues evaluated activity and function before and immediately after MPK provision in individuals with transfemoral amputation and found that self-reported confidence and several clinical functional measures improved, while objectively measured physical activity did not increase [2]. The device changed how walking felt and how patients performed on tests, but not how much they moved in daily life. This is a single study with a short follow-up period immediately after provision, and longer-term activity trajectories may differ. This gap nonetheless invites the central question of this editorial: if the knee itself does not make the leg more active, what does?

Several explanations deserve consideration. Short follow-up may simply be insufficient for behavioral change to emerge; environmental, psychosocial, and comorbidity-related barriers may constrain community ambulation independently of device capability; and habitual activity patterns established over years of prior prosthetic use may be slow to change. The interpretation I develop below -- that the human control loop is a key modifiable factor -- is therefore offered as one contributing explanation among several, and as a hypothesis worth testing rather than an established cause.

Beyond strength: control and coordination

A natural first hypothesis is that residual muscle strength is the missing ingredient. Here the evidence cautions against reductionism. Sawers and Fatone showed that the relationship between hip strength and walking and balance performance differs by amputation level, and that in transfemoral users residual hip strength is only weakly expressed in functional locomotion [3]. Rather than fully recruiting available hip strength through the prosthesis, transfemoral users tend to rely on an intact-limb strategy. These findings are consistent with -- though they do not establish -- the possibility that the limiting factor is frequently not how much strength exists but whether that strength can be voluntarily controlled and coordinated with the device. This reframes the control problem. Advancing the device-side loop -- smarter sensing, faster resistance modulation - - does not remove the need for a human-side loop; it arguably makes the quality of human input more decisive, not less. This is a conceptual argument rather than an empirically demonstrated relationship. Whatever the amputation level, the human remains the origin of control on the account I am proposing here, and the device amplifies good input rather than substituting for it.

Practice: preparing the body before the device

What does a coordination-oriented rehabilitation look like in practice? In a report of two patients with bilateral transfemoral amputation, a staged program was used that began with stubby (shortened) prostheses to establish walking stability at a low center of mass, and introduced MPKs only once the body had acquired the postural control and voluntary coordination to use them well [4]. In these two cases, the staged sequence appeared clinically advantageous, though a two-patient report -- my own -- cannot establish superiority over alternative sequencing. The proposed rationale is that the device was fitted to a body prepared to drive it, not asked to compensate for a body that was not. On this view, investment in device technology and investment in bodily function are not competing priorities but synergistic ones -- each raises the return on the other. A reasonable implication, if this framing is correct, is that clinicians may wish to build the human control loop deliberately, so that when the microprocessor knee arrives, there is a capable operator to meet it.

Embodiment: the role of sustained use

If voluntary control unlocks the device, sustained use may be the path by which the device becomes part of the body. Aizu and colleagues demonstrated a use-dependent increase in attention directed toward the prosthetic foot in people with lower limb amputation, a finding the authors interpret as potentially reflecting gradual integration of the prosthesis into the body schema [5]. Attention allocation is an indirect index, however, and should not be equated with embodiment itself. The proposed principle that sustained use supports embodiment may extend beyond the knee -- to microprocessor feet, hip-level components, and powered limbs -- though this remains a hypothesis rather than a demonstrated finding. This framing also raises a question worth asking: whether over-reliance on the device, letting it do what the user could learn to do, might impede rather than support embodiment. To my knowledge, this has not been directly tested, and it is raised here as a research priority rather than an established risk. The principle that the human controls the leg therefore retains its relevance in the era of intelligent prostheses.

Conclusions

Microprocessor and powered prostheses deliver genuine benefits, but they do not relieve the user of the task of control; if anything, they raise the premium on it. This framework suggests that voluntary control, coordination, and use-driven embodiment merit evaluation as rehabilitation targets alongside device provision -- preparing the body to drive the device, and guarding against a dependence that could undermine the sense of ownership these technologies are intended to support. Whether such an approach improves objective activity, functional outcomes, or embodiment remains an open empirical question, and defining testable outcomes for it is, in my view, a priority for the field. Beyond the microprocessor knee, and beyond the device more generally, the human is the origin of control, as I have argued here.
